# Different Mechanistic Association of Myopia with Rhegmatogenous Retinal Detachment between Young and Elderly Patients

**DOI:** 10.1155/2019/5357241

**Published:** 2019-08-07

**Authors:** Min Seok Kim, Sang Jun Park, Kyu Hyung Park, Se Joon Woo

**Affiliations:** Department of Ophthalmology, Seoul National University College of Medicine, Seoul National University Bundang Hospital, Seongnam, Republic of Korea

## Abstract

**Objectives:**

To investigate the mechanism of rhegmatogenous retinal detachment (RRD) in young and elderly Korean patients based on the results of axial length distribution.

**Subjects/Methods:**

We retrospectively reviewed the medical records of 1599 patients with RRD who had bilateral axial length data examined at one center between 2003 and 2018. Axial lengths were measured using ultrasound or IOLMaster500. The frequency of RRD and axial length distribution according to age groups were investigated.

**Results:**

Patients with RRD displayed a bimodal distribution across ages with two age groups showing the highest peak at 55–59 years and a second peak at 25–29 years of age. The mean axial length was significantly longer in patients younger than 50 years old than that in patients ≥ 50 years old (26.18 ± 1.86 mm vs. 24.55 ± 1.67 mm, respectively, p < 0.001). The percentage of patients with high myopia (axial length ≥ 26 mm) in patients < 50 years old was higher than that in those ≥ 50 years old (51.9% vs. 15.0%, respectively, p < 0.001; odds ratio, 6.11; 95% confidence interval, 4.83 to 7.74).

**Conclusions:**

We found a difference in the prevalence of myopia between young and elderly patients with RRD, which corresponds to a bimodal distribution of RRD incidence in East Asian countries. Our data indicate that myopia or high myopia-induced early vitreous detachment appears to be a major mechanism of occurrence of RRD in young East Asian patients, while senile vitreous liquefaction and detachment is the main mechanism of RRD in elderly patients.

## 1. Introduction

Rhegmatogenous retinal detachment (RRD) is characterized by the separation of the inner neurosensory retina from the outer retinal pigment epithelium resulting from one or more full-thickness retinal breaks. Three essential features are required for the occurrence of RRD: (1) liquefied vitreous gel; (2) traction forces that can produce a retinal break; and (3) the presence of a retinal break that allows passage of liquefied vitreous into the subretinal space [1].

Vitreous syneresis, which induces posterior vitreous detachment, can produce all three features that lead to RRD. Therefore, spontaneous RRD is usually preceded by posterior vitreous detachment. An increasing trend is observed in the prevalence of posterior vitreous detachment with age. In one study, posterior vitreous detachment was observed in 27% of patients aged 60–69 years and 63% of patients over the age of 70 years [2]. Myopia is another risk factor that accelerates posterior vitreous detachment, independently of aging. This myopic vitreous liquefaction could be explained by an increased vitreous volume that exceeds the production of gel components filling the expanding chamber [3]. Thus, aging and myopia have been consistently reported as risk factors for RRD through the mechanism of posterior vitreous detachment.

Our previous nationwide study of RRD in South Korea found that the peak incidence demonstrated a bimodal distribution across age groups [4]. The highest peak, representing patients between 65 and 69 years of age, is due to aging-induced posterior vitreous detachment, while the second peak, representing patients from 20 to 29 years of age, was assumed to be the result of the high prevalence of myopia reported in East Asian countries [4–6]. However, there is no clear evidence that the high incidence of RRD in young East Asians is related to myopia. Therefore, we investigated the distribution of axial lengths in patients with RRD by age group to identify the age-specific association between myopia and RRD.

## 2. Materials and Methods

Institutional Review Board (IRB) approval was obtained from the IRB Seoul National University Bundang Hospital (IRB number: B-1811-504-103) and was conducted in accordance with the tenets of the Declaration of Helsinki.

We performed a retrospective review of the medical records of all consecutive patients diagnosed with RRD at Seoul National University Bundang Hospital from May 2003 to September 2018. Participants for whom axial length measurements in both eyes were available were included in the study. Patients with axial lengths shorter than 20 mm or bilateral differences of 3 mm or more were excluded owing to the possibility of measurement errors of axial length in eyes with RRD.

**Figure 1:**
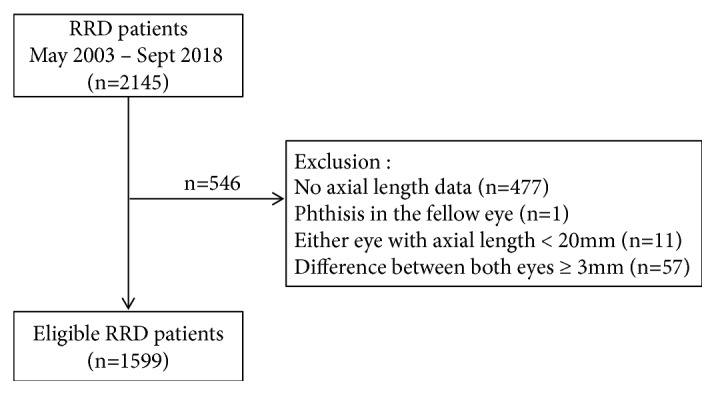
Flow chart showing selection process of enrolled patients with rhegmatogenous retinal detachment (RRD).

During the study period, a total of 2145 cases of RRD were identified. Among these, we excluded 477 patients whose axial length was not measured, one patient with phthisis in the fellow eye (no axial length data in the fellow eye), 11 patients with axial length less than 20 mm, and 57 patients with a difference in bilateral axial lengths of 3 mm or more. Our analysis thus included a total of 1599 patients with bilateral axial length data (Figure 1).

Axial length was measured using the IOL Master 500 (Carl Zeiss Meditec Inc., Jena, Germany) or ultrasound (CineScan, Quantel Medical, Clermont-Ferrand, France) after the diagnosis of RRD as a part of routine examination for surgery preparing against possible complication of cataract. Axial length was measured in both eyes and the mean value was used for subsequent analysis.

## 3. Statistical Analyses

Statistical analyses were performed using SPSS version 25.0 (IBM Corp., Armonk, NY, USA). Pearson's correlation and paired t-tests were used to compare the axial lengths of the RRD eye and the normal fellow eye. Evaluation of the relationship and differences in axial lengths according to age was performed using Pearson's correlation, Student's t-test, and the chi-square test. A p value < 0.05 was considered to indicate significance.

## 4. Results

Of the 1599 included patients, 690 (43%) were women, and the mean age was 49.5 ± 16.9 years (range, 8 to 88 years). The mean axial length was 25.26 ± 1.98 mm in the right eye and 25.26 ± 1.96 mm in the left eye (Table 1). The correlation coefficient for the right and left eyes was 0.929 (p < 0.001), with no significant difference observed between both eyes (p = 0.892).

**Figure 2:**
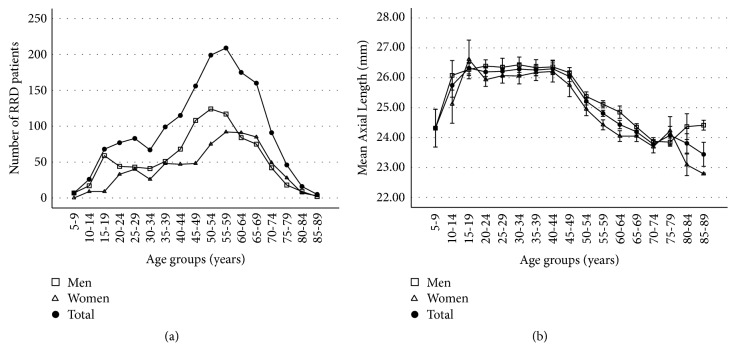
(a) Number of rhegmatogenous retinal detachment (RRD) patients according to age groups during study period from 2003 to 2018. (b) Distribution of mean axial length in RRD patients according to age groups (Error bars: ± 1 standard error).

The distribution of RRD prevalence across the age groups followed a bimodal pattern. Among all participants, the highest peak was found in the group of patients aged 55–59 years, and the second peak was observed in those aged 25–29 years (Table 2, Figure 2(a)).

**Figure 3:**
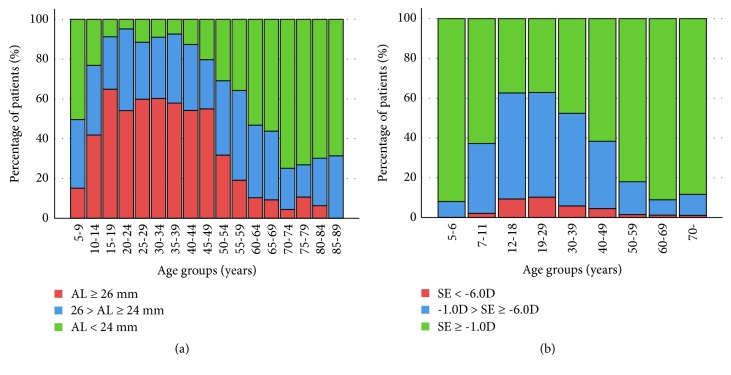
(a) Proportion of patients with different axial length range according to age groups in patients with rhegmatogenous retinal detachment. (b) Percentage of myopic patients in Korea according to age group. Data source: TH Rim et al. Refractive errors in Koreans, 2016 [9]. AL = axial length; SE = spherical equivalent; D = diopter.

The mean axial length was relatively long in patients aged 10–50 years and decreased with age in patients with RRD (Table 2, Figure 2(b)). The mean axial length showed a significant negative correlation with age (r = -0.402, p < 0.001). Two RRD peaks correlating with age and axial length distribution showed a similar pattern between sexes. Patients younger than 50 years old (n = 698) had a significantly longer axial length than that of patients ≥ 50 years old (n = 901) (26.18 ± 1.86 mm vs. 24.55 ± 1.67 mm, respectively, p < 0.001). The percentage of patients with high myopia who had an axial length of 26 mm or longer was higher in individuals younger than 50 years old than in those ≥ 50 years old (51.9% vs. 15.0%, respectively, p < 0.001; odds ratio, 6.11; 95% confidence interval 4.83 to 7.74) (Figure 3(a)).

## 5. Discussion

**Figure 4:**
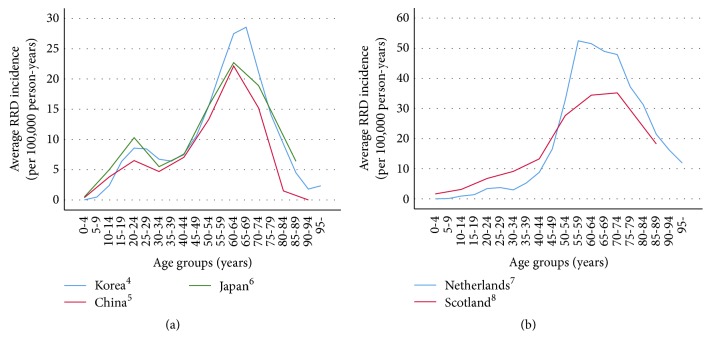
Average annual incidence (per 100,000 person-years) of rhegmatogenous retinal detachment (RRD) in Asian countries (a) and Western countries (b).

In the present study, we found a bimodal distribution of RRD prevalence in 1599 patients across two age groups: one peak in the group aged 55–59 years and a second peak in the group aged 25–29 years. Previous data obtained from the East Asian population support the existence of two similar age-related peaks in RRD incidence [4–6]. The second peak of RRD incidence in the young age group is a typical differential feature of the East Asian population, which has not been observed in the Western population (Figure 4) [7, 8].

If the second peak did not exist, the prevalence of RRD would be low in young individuals and would increase exponentially with age. Thus, the second peak observed in the young age group suggests the presence of another pathogenic mechanism of RRD, in addition to the senile changes in the vitreous gel and vitreoretinal junction. We previously proposed that the high prevalence of myopia and of high myopia in the East Asian population could be a possible mechanism of RRD in the young age group [4]. In the current study, young patients with RRD had relatively longer axial length and demonstrated a higher prevalence of high myopia than the older patients with RRD, supporting our hypothesis that myopia is the main mechanism of RRD in young individuals. Therefore, the graph in Figure 2 suggests that the ages of peak incidence of posterior vitreous detachment and derived retinal tears and RRD are 20s in the high myopes and are 50s to 60s whose eyes are within usual refractive errors.

A nationwide, cross-sectional survey in Korea revealed a prevalence of high myopia (spherical equivalent worse than -6.0 diopters) of 4.5–10.2% in individuals 10–50 years old, decreasing with age (Figure 3(b)) [9]. The prevalence of high myopia (axial length ≥ 26 mm) in patients with RRD in the present study was about 5 times higher than that in individuals of the same age group among the normal Korean population. Although the definition of “high myopia” differed between the two studies, previous studies reported that an axial length around 26 mm corresponds to a refractive error of approximately -6.0 diopters [10–12]. This discrepancy in the prevalence of high myopia between patients with RRD and individuals of the same young age group in the general population strongly suggests that high myopia is a leading mechanism of RRD in young Korean patients.

Myopia is a well-known risk factor for retinal tears and RRD, and the risk increases with higher degrees of myopia [13, 14]. In myopic eyes, the large volume of eyeball induces RRD through the earlier occurrence of posterior vitreous detachment, common lattice degeneration, and thinner retina than that observed in emmetropic eyes [15]. Most ophthalmologists agree that axial length is the strongest determinant of refractive errors [16]. However, “high myopia” does not always indicate “long axial length” because the refractive status of the eye is dependent not only on axial length but also on the optical power of the cornea, aqueous humor, lens, and the vitreous humor [17]. For this reason, we used the axial length data, rather than refractive errors, to accurately reflect the volumes of the eyeball and vitreous cavity in the present study.

As mentioned above, the prevalence of myopia is higher in young than in elderly Koreans. As these young myopic individuals age, they will be exposed to two risk factors for RRD simultaneously: age-induced posterior vitreous detachment and myopia-induced posterior vitreous detachment. In addition, a relatively large middle-aged population and an aging society in Korea are additional risk factors for a rapid increase of RRD incidence in the future [4]. Considering this alarming prediction, clinicians should be alert and should prepare for a large number of patients with RRD in the older age group in the future. A similar situation may occur in other East Asian countries.

The limitations of this study are the retrospective design and the potential selection bias caused by collecting data from only one hospital. However, the large number of patients with RRD and the fact that our data showed a distribution similar to that of previous data from Asian population studies help to balance the limitations. Further studies with large populations of different ethnicities are required to confirm our results. Second, the measurement of axial length in eyes with RRD might have not been accurate. Because of the long enrollment period, ultrasound was replaced as the instrument for axial length measurement by partial coherence laser interferometry (IOL Master). The axial length measured by the optical method was reported to be about 0.39–0.46 mm longer than that measured by ultrasound [18, 19]. However, owing to the large number of patients, the small discrepancy between the measurement methods may not have altered the results. Another possible inconsistency to consider is that there might have been measurement errors of axial length in eyes with RRD. It is ideal to measure the axial length prior to the occurrence of retinal detachment because the axial length value may be inaccurate after the detachment progresses, especially in the case of macular detachment. However, it is unfeasible to measure axial length in eyes in which retinal detachment is expected to occur. To overcome this difficulty, we used the mean values of axial length of bilateral eyes. Indeed, our finding of no significant difference in axial length between bilateral eyes in the present study confirms that there were minimal measurement errors in axial length in diseased eyes.

## 6. Conclusion

In conclusion, young patients with RRD have longer axial length and higher prevalence of high myopia than do elderly patients with RRD. Considering the bimodal incidence of RRD among age groups in East Asian populations, myopia-induced posterior vitreous detachment appears to be a major mechanism of occurrence of RRD in young East Asian patients.

## Figures and Tables

**Table 1:** Baseline characteristics.

Characteristics	Value
Number of patients	1599
Sex (male/female)	909 (57%)/690 (43%)
Age (years)	49.5 ± 16.9
High myopia (AL ≥ 26 mm)	497 (31%)
Mean AL (mm)	
Right	25.26 ± 1.98
Left	25.26 ± 1.96
Mean	25.26 ± 1.93

SD = standard deviation.

AL = axial length.

**Table 2:** Distribution of RRD patients and axial length according to age group.

Age group (years)	Number (%)				Mean AL (Mean ± SD)		
	Total	Male	Female	AL ≥ 26 mm	Total	Male	Female
5–9	7 (0.4)	7	0	1 (14.3)	24.32 ± 1.67	24.32 ± 1.67	NA
10–14	26 (1.6)	17	9	10 (38.5)	25.75 ± 2.01	26.08 ± 2.04	25.12 ± 1.91
15–19	68 (4.3)	59	9	41 (60.3)	26.32 ± 1.61	26.28 ± 1.56	26.61 ± 1.94
20–24	77 (4.8)	44	33	37 (48.1)	26.20 ± 1.36	26.39 ± 1.39	25.94 ± 1.29
25–29	83 (5.2)	43	40	46 (55.4)	26.22 ± 1.78	26.35 ± 1.97	26.07 ± 1.57
30–34	67 (4.2)	41	26	37 (55.2)	26.29 ± 1.54	26.44 ± 1.64	26.06 ± 1.35
35–39	99 (6.2)	51	48	52 (52.5)	26.26 ± 1.89	26.34 ± 1.92	26.18 ± 1.87
40–44	115 (7.2)	68	47	57 (49.6)	26.30 ± 2.10	26.36 ± 1.86	26.21 ± 2.42
45–49	156 (9.8)	108	48	81 (51.9)	26.04 ± 2.10	26.17 ± 1.80	25.75 ± 2.65
50–54	199 (12.4)	124	75	58 (29.1)	25.22 ± 1.77	25.38 ± 1.69	24.95 ± 1.89
55–59	209 (13.1)	117	92	36 (17.2)	24.81 ± 1.48	25.11 ± 1.30	24.43 ± 1.61
60–64	175 (10.9)	84	91	17 (9.7)	24.44 ± 1.85	24.85 ± 1.91	24.05 ± 1.71
65–69	160 (10.0)	75	85	14 (8.8)	24.19 ± 1.39	24.35 ± 0.97	24.05 ± 1.67
70–74	91 (5.7)	42	49	4 (4.4)	23.78 ± 1.19	23.87 ± 0.80	23.70 ± 1.45
75–79	46 (2.9)	18	28	5 (10.9)	24.08 ± 1.99	23.85 ± 0.58	24.22 ± 2.51
80–84	16 (1.0)	9	7	1 (6.3)	23.81 ± 1.30	24.37 ± 1.29	23.09 ± 0.96
85–89	5 (0.3)	2	3	0 (0)	23.44 ± 0.90	24.42 ± 0.23	22.79 ± 0.05

Total	1599	909	690	497	25.26 ± 1.93	25.53 ± 1.79	24.91 ± 2.06
	(100)	(56.8)	(43.2)				

RRD = rhegmatogenous retinal detachment.

AL = axial length.

SD = standard deviation.

## Data Availability

The data used to support the findings of this study are available from the corresponding author upon request.
